# Supplementary figures and images for: Highly sensitive molecular assay based on Identical Multi-Repeat Sequence (IMRS) algorithm for the detection of Trichomonas vaginalis infection
Source: PLoS One. 2025 Feb 7;20(2):e0317958. doi: 10.1371/journal.pone.0317958 (PMC11805422; doi:10.1371/journal.pone.0317958)

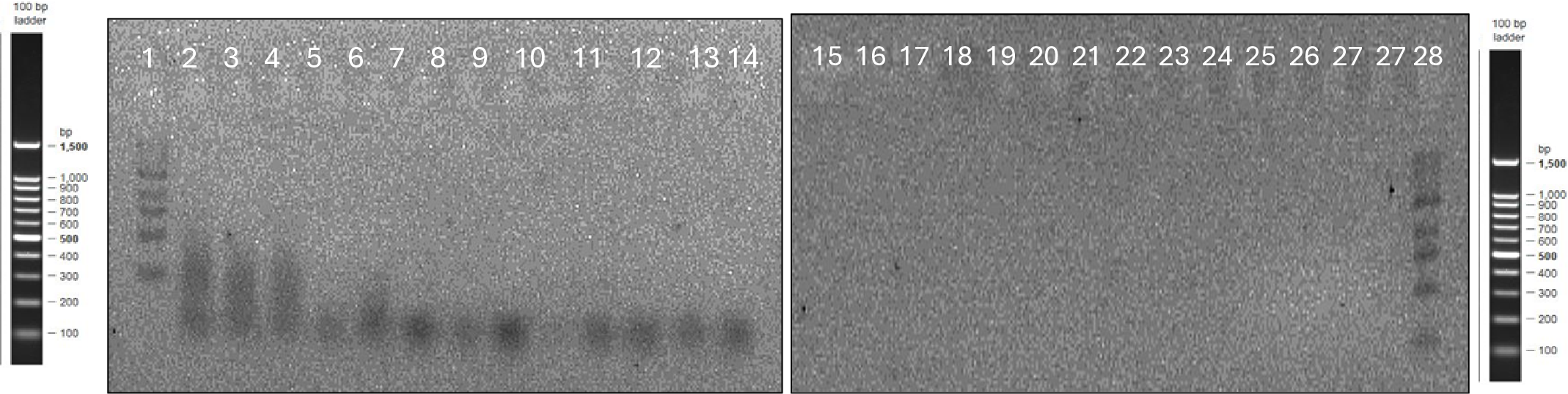

Supplement: S1 Fig — (TIF) [file pone.0317958.s001.tif]

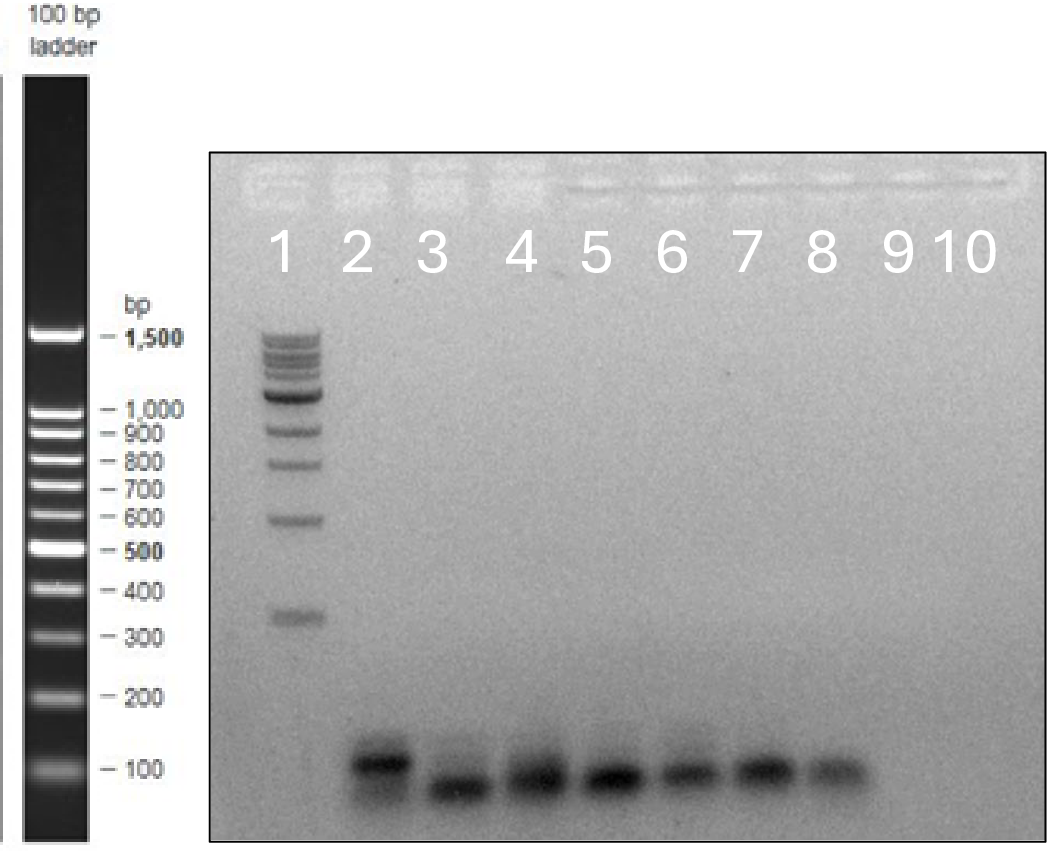

Supplement: S2 Fig — (TIF) [file pone.0317958.s002.tif]

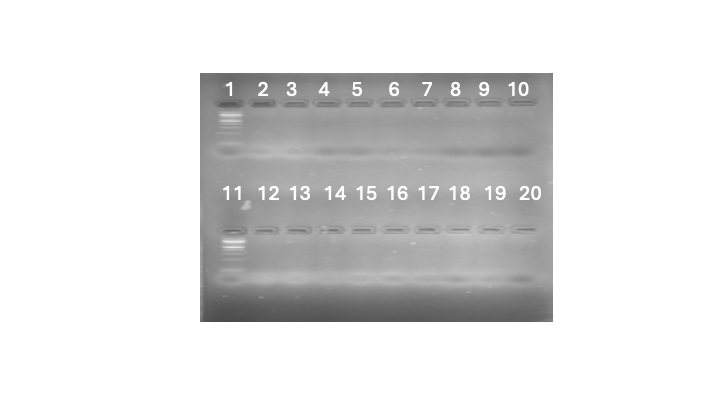

Supplement: S3 Fig — Well number 1 and 11 is 100bp ladder. (TIFF) [file pone.0317958.s003.tiff]
